# Familial Kaposi's Sarcoma: A Report of Five Cases from Greece

**DOI:** 10.1155/2014/671631

**Published:** 2014-06-29

**Authors:** Kalliopi Armyra, Anargyros Kouris, Arsinoi Xanthinaki, Alexandros Stratigos, Irene Potouridou

**Affiliations:** Department of Dermatology and Venereology, Hospital “Andreas Sygros”, Ionos Dragoumi 5, 16121 Athens, Greece

## Abstract

*Introduction*. Familial cases of Kaposi's sarcoma have rarely been reported. Kaposi's sarcoma is not uncommon in Greece; its incidence is estimated at 0.20 per 100.000 habitants, showing an increased predominance in the Peloponnese, in Southern Greece. *Case Report*. We describe five cases of familial clustering of KS originating from Greece. *Discussion*. The pathogenesis of familial Kaposi's sarcoma is still far from being completely understood. Genetic, environmental, and infectious factors have been incriminated.

## 1. Introduction

Kaposi's sarcoma (KS) is a multifocal disease that was first described in 1872 by Moritz Kaposi. It has four principal clinical variants: (1) classic KS, (2) African endemic KS, (3) KS in iatrogenically immunocompromised patients, and (4) AIDS-related epidemic KS. Classic KS presents as blue-red to violet macules on the distal lower extremities that coalesce to form large plaques or develop into nodules or polypoid tumors. Initially, unilateral lesions may progress to a more widely disseminated multifocal pattern. Early lesions may regress, while others evolve and lead to lesions at different stages. Patients may also have lesions in the mouth and gastrointestinal tract that are usually asymptomatic.

Classic Kaposi's sarcoma mainly affects men over the age of 50 years, generally Jewish or of Mediterranean/Eastern Europe descent, and is also known as “Mediterranean KS.” The highest incidence is observed in the Mediterranean area, especially in Sardinia and Southern Italy [1]. In Italy, the incidence is reported to be 0.98 per 100.000 men and 0.41 per 100.000 women [1]. In Greece, it is estimated at 0.20 per 100.000 habitants [2].

Although familial occurrence is rare, clustered occurrences within families have been reported in Sardinia and in the Peloponnese, in Southern Greece [3]. Familial clustering of classic KS suggests that infectious (viral), environmental, and genetic factors, either independently or in combination, contribute to the pathogenesis of the disease.

This is a retrospective study that reports five families, each of which had two members affected by KS. The patients were HIV-negative and had no recognized underlying immunodeficiency (Table 1). Four of the five familial occurrences originated from the Peloponnese and one from Central Greece who lived in Athens.

## 2. Case Reports

### 2.1. Family 1

**Figure 1:**
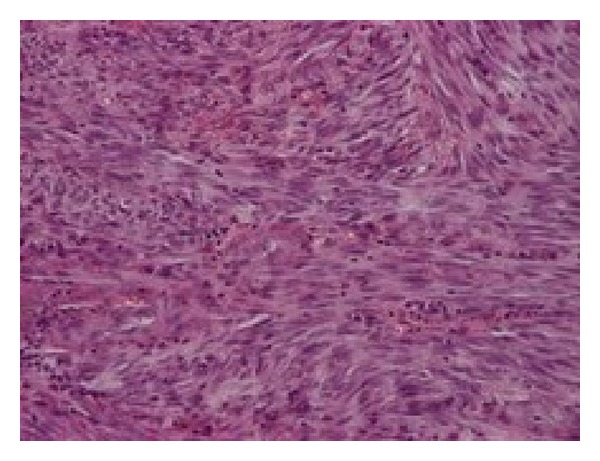
Spindle shaped forming numerous vascular slit-like spaces filled by red blood cells. (H+E ×250).

A 78-year-old man, who originated from Southern Greece, was referred to “A. Sygros” Hospital exhibiting reddish-brown cutaneous lesions over different parts of his body that had manifested 18 years earlier. A biopsy specimen from lesional skin confirmed the clinical diagnosis of KS (Figure 1). The patient was treated with subcutaneous recombinant interferon alpha-2a (3 × 10^6^ IU, 3 times per week). After one year, the dose was reduced to 3 × 10^6^ IU once per month. At the last follow-up visit, all lesions displayed a marked decrease in the size.

**Figure 2:**
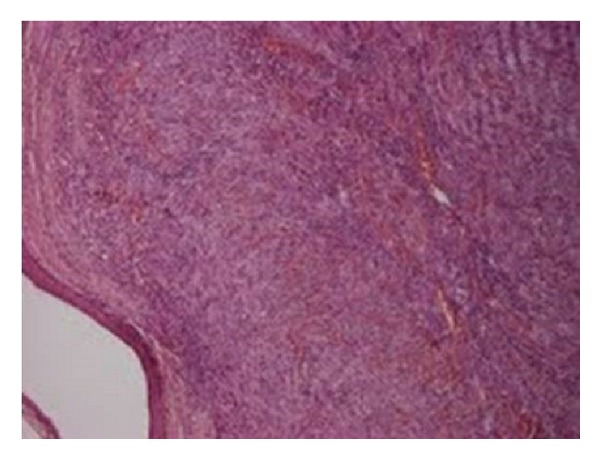
Blood filled vascular spaces-slits closely associated with interwearing spindle cells. (H+E ×40).

A 36-year-old heterosexual man, son of the above mentioned patient, visited the outpatient clinic of “A. Sygros” Hospital with a red lesion that had appeared on his upper and lower extremities three years earlier. A skin biopsy indicated Kaposi's sarcoma (Figure 2). The patient was treated with alitretinoin gel 0.1%. At the scheduled follow-up visit, one month later, the improvement was visible.

### 2.2. Family 2

**Figure 3:**
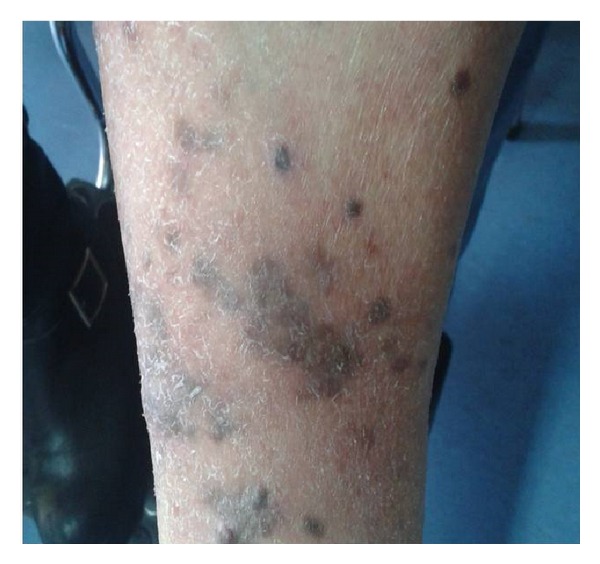
Reddish-violaceous papules, nodules, and infiltrated plaques on the legs.

A 72-year-old woman was admitted to “A. Sygros” Hospital with multiple violaceous cutaneous lesions all over her body (Figure 3). The disease had started one year earlier, with the appearance of a few purple macules and papules on her knees. The biopsy revealed Kaposi's sarcoma. The patient was treated with subcutaneous recombinant interferon alpha-2a (3 × 10^6^ IU three times per week). Partial remission was observed 6 months later.

The son of the above patient, at the age of 51, was referred to “A. Sygros” Hospital with reddish cutaneous lesions in his left abdominal area. Biopsy specimens demonstrated Kaposi's sarcoma. Alitretinoin gel 0.1% was administered. The patient returned two years later with disseminated cutaneous KS. The patient was treated with doxorubicin pegylated 20 mg/m^2^. After 6 cycles, almost complete remission of the disease was achieved. Both patients came from the Peloponnese, Southern Greece.

### 2.3. Family 3

A 72-year-old female, who originated from Central Greece, was admitted to our hospital for treatment of recurrent Kaposi's sarcoma. The disease had started 3 years earlier, at the age of 69, with the appearance of a few violaceous macules and papules on the right lateral malleolus area. The lesions were diagnosed as KS and treated with radiotherapy. Six months before admission to our hospital, she displayed a recurrence of the disease. Localized radiotherapy resulted in remission of the lesions. At the scheduled follow-up visit, six months later, the lesions displayed noticeable improvement.

**Figure 4:**
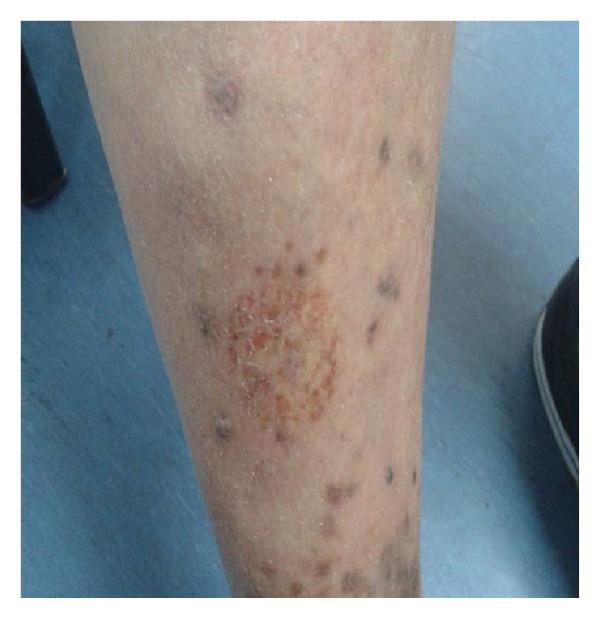
Reddish-purple lesions on the legs.

Her sister, aged 51, visited the outpatient clinic of “A. Sygros” Hospital with reddish-purple lesions on her hands and on the legs (Figure 4). Topical alitretinoin gel 0.1% was administered. At the last follow-up visit, all lesions showed a marked decrease in size.

### 2.4. Family 4

A 65-year-old man was referred to “A. Sygros” Hospital with reddish-brown cutaneous lesions over different parts of his body. The lesions had developed 8 months earlier, first appearing on his left thigh and gradually spreading to other parts of his body. The lesions were treated with cryosurgery. At the scheduled follow-up visit, three months later, the patient displayed a relapse and was treated with alitretinoin gel 0.1%. By the last follow-up visit, there was a marked decrease in five of seven lesions.

His brother, aged 79, visited the outpatient clinic of “A. Sygros” Hospital with a few purple macules and papules on his lower extremities. Clinical examination revealed several reddish-purple macules and a biopsy indicated Kaposi's sarcoma. The patient was treated with subcutaneous recombinant interferon alpha-2a (3 × 10^6^ IU, 3 times a week) for one year. After two months of follow-up, lesions were markedly decreased. Both patients originated from the Peloponnese, Southern Greece.

### 2.5. Family 5

A 75-year-old man, who originated from the Peloponnese, was referred to “A. Sygros” Hospital with reddish-brown cutaneous lesions on his legs. The lesions had developed two years earlier, first appearing on his left thigh and right knee. The lesions were treated with alitretinoin gel 0.1%. At the last follow-up visit, there was a marked decrease in all lesions.

His son, aged 57, visited the outpatient clinic of “A. Sygros” Hospital with reddish-purple lesions on his left hand and on the legs. The lesions were treated with cryosurgery. Partial remission was observed 5 months later.

## 3. Discussion

Classic Kaposi's sarcoma is not uncommon in Greece. There are sporadic cases all over the country; endemic clustering has been observed in southern regions. A study from January 1990 to December 1994 estimated an incidence of 2.11 cases of KS per 100000 habitants, representing 1.35% of all malignancies [4]. In the Peloponnese, the incidence is estimated to be 0.8 per 100000 while, in more restricted areas of the Southern Peloponnese, it is 3-4 times higher, approximating that of African Kaposi's sarcoma [2]. Familial cases of KS have been reported in Greece previously [4, 5]. This is the third report of familial Kaposi sarcoma in Greece to be documented.

The first familial case was reported in 1909 by Radaeli [6]; since then, only a few cases have been published in the literature [5–10]. Finlay and Marks described a case of Kaposi's sarcoma in a mother and son of Italian origin [7]. Perniciaro et al. reported a case of a brother and sister, who were of German/English descent and suffered from KS on the lower extremity [8]. Cottoni et al. cited four families with KS, occurring in uncle and nephew, father and son, and two pairs of brothers, originating from Sardinia [9]. Guttman-Yassky et al. described a rare case of four Jewish siblings suffering from classic Kaposi's sarcoma [10].

Familial cases suggest that hereditary factors are involved in the pathogenesis of Kaposi's sarcoma. Different HLA antigens could affect individual susceptibility to the disease. Immunogenetic studies have shown an increased incidence of HLA DR5 antigen in familial Kaposi's sarcoma [9]. Guttmann-Yassky et al. analyzed 8 family members and demonstrated that 7 of them shared the HLA DRB1∗11 antigen [10]. In a study of 32 Greek patients, an increased frequency of HLA-B18 and HLA-DR5 was demonstrated [11].

Classic Kaposi's sarcoma seems to be associated with human herpes virus 8 (HHV8) or Kaposi's sarcoma-associated herpes virus (KSHV). The prevalence of KSHV varies among geographic regions from 2% to 7% in Western Europe and North America, from 10% to 20% in the Mediterranean, and up to 100% in Sub-Saharan African countries [12]. KSHV infection is asymptomatic in most infected individuals. Nevertheless, it can provoke KS, albeit its progression is very slow. Classic KS develops in 0.03%–0.05% of individuals infected by KSHV aged over 50 years [13]. It is similarly observed that KSHV infection is higher among family members of cKS patients than that observed in control studies, which indicates intrafamiliar transmission of KSHV [1, 14]. In our retrospective study, some patients have passed away, while others are not followed up by our department anymore. This is the reason why we could not test KSHV.

Familial clustering of KS is rare and this fact argues against simple Mendelian inheritance. Nevertheless, the possibility that complex predisposing factors are involved cannot be excluded [10]. We presented five families, each having two members presenting with classic Kaposi's sarcoma. An interaction between a malignancy-linked virus, KSHV, genetic host factors, and altered immunity could possibly cause this disease. Further genetic investigation in larger studies is needed to elucidate whether there is a predisposition to infection or tumor formation within families of classic KS patients. Identifying risk factors of familial KS has important implications in the prevention and therapeutic approaches of this tumor.

## Figures and Tables

**Table 1:** Characteristics of the patients with KS.

Family	Relationship	Age at the time of diagnosis	Age of onset	Type of KS	Location of the lesion	Duration (years)	Extracutaneous involvement	Risk factors	Treatment
1	Father	78	60	Reddish-brown macules and reddish-violaceous infiltrated plaques	Right thigh, calves, feet (including the toes), and hands	18	None	Diabetes mellitus type II	Interferon A-2a sc
	Son	36	33	Reddish-purple macules and irregular plaques	Thighs, knees, left forearm, the area of the right achilles tendon, and the middle finger of the left hand	3	None	None	Gel alitretinoin 0.1%

2	Mother	72	71	Reddish-violaceous papules, nodules, and infiltrated plaques	Chest, abdomen, back, and both arms and legs	1	None	None	Interferon A-2a sc
	Son	51	51	Reddish-brown papules and nodules	Abdominal area, right chest wall, left buttock, both thighs, both the medial malleoli areas, and the toes of both feet	1	None	Diabetes mellitus type II	Gel alitretinoin 0.1%, doxorubicin pegylated 20 mg/m^2^

3	Sister	72	69	Reddish-violaceous papules and nodules	Right lateral, medial malleolus area	3	None	None	Radiotherapy
	Sister	51	51	Reddish-purple nodules and infiltrating plaques	Both hands, knees, calves, and feet	3 months	None	None	Gel alitretinoin 0.1%

4	Brother	65	65	Reddish-purple macules and irregular plaques	Both thighs, knees, and the right forearm	8 months	None	None	Cryosurgery, gel alitretinoin 0.1%
	Brother	79	79	Reddish-purple macules	Lower extremities	7 months	None	Diabetes mellitus type II, hypertension	Interferon A-2a sc

5	Father	75	73	Reddish-purple macules and irregular plaques	Both legs	2	None	Diabetes mellitus type II	Gel alitretinoin 0.1%
	Son	57	57	Reddish-purple nodules and infiltrating plaques	Left hand and knees	3 months	None	None	Cryosurgery

## References

[1] Mancuso R, Brambilla L, Agostini S (2011). Intrafamiliar transmission of Kaposi's sarcomaassociated herpesvirus and seronegative infection in family members of classic Kaposi's sarcoma patients. *Journal of General Virology*.

[2] Kaloterakis A (1984). *Kaposi’s sarcoma in Greece. Mediterranean Kaposi’s sarcoma [PhD. Thesis]*.

[3] Rappersberger K, Tschachler E, Zonzits E (1990). Endemic Kaposi's sarcoma in human immunodeficiency virus type 1-seronegative persons: Demonstration of retrovirus-like particles in cutaneous lesions. *Journal of Investigative Dermatology*.

[4] Stratigos JD, Potouridou I, Katoulis AC (1997). Classic Kaposi's sarcoma in Greece: a clinico-epidemiological profile. *International Journal of Dermatology*.

[5] Kaloterakis A, Papasteriades C, Filiotou A, Economidou J, Hadjiyannis S, Stratigos J (1995). HLA in familial and nonfamilial Mediterranean Kaposi's sarcoma in Greece. *Tissue Antigens*.

[6] Radaeli F (1909). Nuovo contributo alla conoscenza dell’angioendotelioma cutaneo (Sarcoma idiopatico multiplo) di Kaposi. *Giorn. Ital. della Mal. Ven. e della Pelle*.

[7] Finlay AY, Marks R (1979). Familial Kaposi’s sarcoma. *British Journal of Dermatology*.

[8] Perniciaro C, Gross DJ, White JW, Adrian RM (1996). Familial Kaposi's sarcoma. *Cutis*.

[9] Cottoni F, Masia IM, Masala MV, Mulargia M, Contu L (1996). Familial Kaposi's sarcoma: case reports and review of the literature. *Acta Dermato-Venereologica*.

[10] Guttman-Yassky E, Cohen A, Kra-Oz Z (2004). Familial clustering of classic Kaposi sarcoma. *The Journal of Infectious Diseases*.

[11] Papasteriades C, Kaloterakis A, Filiotou A (1984). Histocompatibility antigens HLA-A, -B, -DR in Greek patients with Kaposi's sarcoma. *Tissue Antigens*.

[12] Schulz TF (1999). Epidemiology of Kaposi's sarcoma-associated herpesvirus/human herpesvirus 8. *Advances in Cancer Research*.

[13] Vitale F, Briffa DV, Whitby D (2001). Kaposi's sarcoma herpes virus and Kaposi's sarcoma in the elderly populations of 3 Mediterranean islands. *International Journal of Cancer*.

[14] Guttman-Yassky E, Kra-Oz Z, Dubnov J (2005). Infection with Kaposi's sarcoma-associated herpesvirus among families of patients with Classic Kaposi's sarcoma. *Archives of Dermatology*.

